# Editorial: Global excellence in cardiovascular medicine: Central and South America

**DOI:** 10.3389/fcvm.2024.1429182

**Published:** 2024-06-17

**Authors:** Patricio Lopez-Jaramillo

**Affiliations:** Masira Research Institute, Medical School, Universidad de Santander (UDES), Bucaramanga, Colombia and Facultad de Ciencias Médicas Eugenio Espejo, Universidad UTE, Quito, Ecuador

**Keywords:** Central and South America, cardiovascular risk factors, hypertension, waist circumference, air pollution

**Editorial on the Research Topic** Global excellence in cardiovascular medicine: Central and South America

In the last decades Central and South America have experienced rapid changes in lifestyle resulting in a higher burden of cardiometabolic risk factors and a greater contribution of non-communicable diseases to mortality and morbidity (1). Recently, the results of a large prospective study conducted in South America showed that over two-thirds of deaths in the region were due to either cardiovascular disease (CVD), cancer, or respiratory diseases. Only modest differences in the incidence of CVD were observed between countries (2). In all countries, men experienced higher rates of CVD and death compared with women. Deaths were also consistently higher in rural areas across all countries. Incident CVD was largely attributable to 12 modifiable risk factors, with metabolic risk factors being the most prevalent. The largest population-attributable fractions (PAF) for CVD were hypertension, smoking, and abdominal obesity. Outdoor air pollution, an important risk factor for CVD (3), was not included.

The present issue of Frontiers in Cardiovascular Medicine focuses on Latin America and publishes eight articles from groups working in Brazil, Chile, Colombia, and Peru.

Hypertension is the risk factor more studied and sought from different points of view by the Latin American researchers. Amorim et al. compared the sociodemographic, therapeutic, and anthropometric characteristics of 2.956 Brazilian hypertensive patients attending public and private centers, which showed 67.8% of uncontrolled hypertension in the public centers with some improvement in the private centers (47.6%). These data are similar to those previously reported for all South American countries (4). These results highlight the need to implement new strategies to improve hypertensive control in the region as demonstrated by a community-based comprehensive intervention (5).

As Latin America is a region of enormous geographical and ethnic differences, with the countries in the Southern Cone experiencing different seasons, Barbosa et al. reviewed the impact of seasonal variations of blood pressure on hypertension phenotypes and the role of air pollution, latitude, and altitude. The authors conclude that in general, but particularly in our region, cohort studies with large samples that include populations with different socio-economic, ethnic and cultural backgrounds are lacking to explain the crucial relationship between environmental factors and hypertension. This definition is important because it has been reported that in Afro-descendant individuals the higher prevalence of hypertension is mediated by the level of education, a parameter indicator of socioeconomic status (6).

Lanas et al. in Temuco, a Southern Chilean city with elevated levels of air pollution using wood as a combustible during the cold season, reported that the season of high air pollution was associated with increased cardiometabolic risk factors and estimated cardiovascular risk, regardless of the lower levels of circulating acute inflammatory molecules. This contradiction is explained by the authors as the presence of additional causal factors related to lifestyle during the cold season. Public health policies to control air pollution and improve the control of traditional risk factors should be implemented to reduce the significant increase in cardiovascular events during the cold season.

Hypertension is an important risk factor for cerebrovascular disease (7). Measurement of intracranial pressure may be useful in evaluating the ability of cerebral autoregulation and vascular barriers to protect the brain. Costa et al. presented interesting results of measuring intracranial pressure waveform in 391 long-term essential hypertensive patients with a new non-invasive device that could detect and monitor nanometric skull bone displacement for each cardiac cycle. Normal intracranial pressure was observed in 21.7% of patients, intracranial compliance disorder in 32.7% and intracranial hypertension in 45.6%. These results suggest that the non-invasive device developed and validated by the authors is a safe and precise measurement of intracranial pressure that could contribute to a better understanding of the brain damage induced by hypertension.

Guimaraes Filho et al., presented results showing that in 59 patients the treatment of hypertension guided by central pressure reduction goals, rather than peripheral blood pressure measurement was not able to demonstrate differences in outcomes related to pulse wave velocity and target organ damage, but showed superiority in reducing central diastolic pressure and AIx behavior at the end of a one-year follow-up, opening the possibility that longer follow-ups and greater sampling power may demonstrate the benefits of this treatment strategy.

Pereira et al. evaluated the best cutoff SAGE score, a score based on four clinical parameters (peripheral systolic blood pressure, age, fasting glucose and glomerular filtration rate), that would indicate the risk of a pulse wave velocity ≥10 m/s, a predictor of cardiovascular events, in 212 Brazilian hypertensive patients. The best SAGE score was ≥7 which is a good predictor and a useful tool for the identification of Brazilian hypertensive patients with elevated pulse wave velocity.

The article by Lopez-Lopez et al. contributed to resolving an old controversy about the waist circumference cut-off point that best identifies the risk of major cardiovascular events (MACE) and incident diabetes in Colombia (8, 9). Data from 6.580 participants in the PURE study cohort were analyzed, after a mean follow-up of 12 years. There were 635 cases of MACE and incident diabetes. A cut-off of 89 cm for men and 86 cm for women are the more sensitive values to identify elevated risks of MACE and incident diabetes. These values were associated with a 1.76-fold and 1.41-fold increased risk of presenting the composite outcome in men and women respectively. The authors suggest using these cut-off points to assess cardiovascular risk in Latin America, but we are expecting the report that includes the other South American countries participating in the PURE study to see if these cut-off points are similar throughout the region, considering that lifestyle changes have started at different times in these countries and considering the important ethnic differences that exist.

Finally, Velarde-Acosta et al., reported the case of a 54-year-old female patient with Takotsubo syndrome (TTS), a rare cardiomyopathy whose pathophysiology is probably linked to an excess of catecholamines causing cardiac stunning and transient ventricular systolic dysfunction. Initially the patient was admitted to the emergency service due to very severe oppressive chest pain associated with nausea, vomiting and sweating. The ECG suggested an acute myocardial infarction, but the coronary angiography did not confirm this suspicion. Cardiac computed tomography showed an intracavitary mass that supported the hypothesis of a myxoma as a trigger of TTS, which was confirmed after the removal of the tumor. The patient remains asymptomatic at 6 weeks of follow-up.

We believe that this issue of Frontiers in Cardiovascular Medicine dedicated to Latin American countries is a good representation of the research that is developing in this area. However, we think that there is much more academic production and that we must insist on inviting our colleagues to participate in this initiative that gives visibility to our work and contributes to define the best strategies to improve the control of the principal risk factors in our region.

## References

[1] Lopez-Jaramillo P Lopez-Lopez J Cohen D Alarcon-Ariza N Mogollon-Zehr M. Epidemiology of hypertension and diabetes mellitus in Latin America. Curr Hypertens Rev. (2021) 17:112–20. 10.2174/1573402116999200917152952 32942979

[2] Lopez-Jaramillo P Joseph P Lopez-Lopez JP Lanas F Avezum A Diaz R Risk factors, cardiovascular disease, and mortality in South America: a PURE substudy. Eur Heart J. (2022) 43:2841–51. 10.1093/eurheartj/ehac113 35325078

[3] Wang Y Duong M Brauer M Rangarajan S Dans A Lanas F Household air pollution and adult lung function change, respiratory disease, and mortality across eleven low- and middle-income countries from the PURE study. Environ Health Perspect. (2023) 13:47015. 10.1289/EHP11179 PMC10132780 37126654

[4] Lamelas P Diaz R Orlandini A Avezum A Oliveira G Mattos A Prevalence, awareness, treatment, and control of hypertension in rural and urban communities in Latin American countries. J Hypertens. (2019) 37:1813–21. 10.1097/HJH.0000000000002108 30964825

[5] Schwalm JD McCready T Lopez-Jaramillo P Yusoff K Attaran A Lamelas P A community-based comprehensive intervention to reduce cardiovascular risk in hypertension (HOPE 4): a cluster-randomised controlled trial. Lancet. (2019) 394:1231–42. 10.1016/S0140-6736(19)31949-X 31488369

[6] Lopez-Lopez JP Cohen DD Alarcon-Ariza N Mogollon-Zehr M Ney-Salazar D Chacon-Manosalva MA Ethnic differences in the prevalence of hypertension in Colombia: association with education level. Am J Hypertens. (2022) 35:610–8. 10.1093/ajh/hpac051 35437579 PMC9248921

[7] O'Donnell MJ Xavier D Liu L Zhang H Chin SL Rao-Melacini P Risk factors for ischaemic and intracerebral haemorrhagic stroke in 22 countries (the INTERSTROKE study): a case-control study. Lancet. (2010) 376:112–23. 10.1016/S0140-6736(10)60834-3 20561675

[8] López-Jaramillo P Rueda-Clausen CF Silva FA. The utility of different definitions of metabolic syndrome in andean population. Int J Cardiol. (2007) 116:421–2. 10.1016/j.ijcard.2006.03.074 16893577

[9] Perez M Casas JP Cubillos-Garzón LA Serrano NC Silva F Morillo CA Using waist circumference as a screening tool to identify Colombian subjects at cardiovascular risk. Eur J Cardiovasc Prev Rehabil. (2003) 10:328–35. 10.1097/01.hjr.0000095050.46631.6f 14663294

